# Association between Mortality and Sequential Organ Failure Assessment Score during a Short Stay in the Intensive Care Unit after Non-Cardiac Surgery

**DOI:** 10.3390/jcm11195865

**Published:** 2022-10-04

**Authors:** Ah Ran Oh, Jungchan Park, Jong-Hwan Lee, Dan-Cheong Choi, Kwangmo Yang, Jin-ho Choi, Joonghyun Ahn, Ji Dong Sung, Seunghwa Lee

**Affiliations:** 1 Department of Anesthesiology and Pain Medicine, Samsung Medical Center, Sungkyunkwan University School of Medicine, Seoul 06351, Korea; 2 Department of Biomedical Sciences, Ajou University Graduate School of Medicine, Suwon 16499, Korea; 3 Center for Health Promotion, Samsung Medical Center, Sungkyunkwan University School of Medicine, Seoul 06351, Korea; 4 Department of Emergency Medicine, Samsung Medical Center, Sungkyunkwan University School of Medicine, Seoul 06351, Korea; 5 Statistics and Data Center, Research Institute for Future Medicine, Samsung Medical Center, Seoul 06351, Korea; 6 Rehabilitation and Prevention Center, Heart Vascular Stroke Institute, Samsung Medical Center, Sungkyunkwan University School of Medicine, Seoul 06351, Korea; 7 Department of Biomedical Engineering, Seoul National University College of Medicine, Seoul 07061, Korea

**Keywords:** intensive care units, mortality, non-cardiac surgery, sequential organ failure assessment score

## Abstract

Background: The sequential organ failure assessment (SOFA) score has been validated in various clinical situations. However, it has not been investigated during a short stay in the intensive care unit (ICU). This study aimed to evaluate the association between the SOFA score and outcomes in patients who were monitored for less than one day after non-cardiac surgery. Methods: From a total of 203,787 consecutive adult patients who underwent non-cardiac surgery between January 2011 and June 2019, we selected 17,714 who were transferred to the ICU immediately after surgery and stayed for less than 24 h. Patients were divided according to quartile value and change between the initial and follow-up levels of SOFA score. Results: Three-year mortality tended to increase with a higher initial SOFA score (11.7%, 11.8%, 15.1%, and 17.8%, respectively). The patients were divided according to changes in the SOFA score at the midnight postoperative follow-up check: 16,176 (91.3%) in the stable group and 1538 (8.7%) in the worsened group. The worsened group showed significantly higher three-year mortality and complications (13.2% vs. 18.6%; HR [hazard ratio]: 1.236; 95% CI [confidence interval]: 1.108–1.402; *p* ≤ 0.0021 for three-year mortality and 3.8% vs. 9.1%; HR: 2.13; 95% CI: 1.73–2.60; *p* < 0.001 for acute kidney injury). Conclusions: The SOFA score during a short stay in the ICU after non-cardiac surgery showed an association with mortality. The change in SOFA score may need to be considered at discharge from the ICU.

## 1. Introduction

Worldwide, more than 300 million major non-cardiac surgeries are performed yearly and are being increasingly offered to an older and more comorbid population [1]. Because these patients carry a certain risk of adverse events after surgery, they are often admitted to the intensive care unit (ICU) for postoperative monitoring [2]. In the group at low risk of postoperative ICU admission, the duration is typically shorter than one day, and ICU-specific treatment is rarely required. These are considered routine monitoring precautions and draw relatively little attention despite the risk of organ dysfunction due to pre-existing organ susceptibility and surgical inflammation. Moreover, short stays of <24 h in the ICU have usually been excluded from outcome studies, as the currently available scoring systems for mortality prediction require at least 24 h of data [3,4,5]. Therefore, there is a paucity of data to evaluate whether it is safe to discharge the patient from the ICU following a short routine monitoring period after surgery. However, given that these patients are similarly exposed to risk factors of mortality such as organ failure due to pre-existing organ susceptibility or surgical inflammation [6], a reliable tool is clinically needed.

The sequential organ failure assessment (SOFA) score is a simple and objective scoring system that represents the degree and progression of organ dysfunction with a four-point assessment for each of the six organ systems (central nervous system, cardiovascular system, respiratory system, renal system, liver, and coagulation) [7]. This assessment was initially developed to describe and quantify organ function and has been validated in various clinical situations. An increase in the SOFA score during ICU treatment has also been associated with progressive organ failure and increased mortality in various clinical settings [8,9,10,11]. However, the suitability of the SOFA score for predicting postoperative outcomes in patients after a short stay in the ICU has never been investigated. In this study, we hypothesized that the predictive value of the SOFA score could be reproduced for outcomes of low-risk patients considering that they share multiorgan dysfunction as a major cause of death [6]. We aimed to determine the association between the SOFA score and postoperative outcomes in patients who stayed in the ICU for less than one day after non-cardiac surgery. Our findings may provide clinical information that can be used to evaluate the safety of discharge after a short ICU stay after non-cardiac surgery.

## 2. Materials and Methods

The registry for this study was curated in de-identified form, and the Institutional Review Board at our institution approved this study and granted a waiver to require written informed consent from individual patients for this study (SMC 2021-06-078). We conducted this study according to the Declaration of Helsinki and reported the results following the Strengthening the Reporting of Observational Studies in Epidemiology guidelines.

### 2.1. Data Curation and Study Population

We generated the SMC-NoCop (Samsung Medical Center-Non Cardiac operation, KCT 0006363) registry using data from 203,787 consecutive adult patients who underwent non-cardiac surgery at the Samsung Medical Center in Seoul, Korea, between January 2011 and June 2019. This large, single-center, de-identified cohort was extracted from the institutional electronic archive system using the “Clinical Data Warehouse Darwin-C” system, which is an electronic system designed to allow investigators to search for and retrieve data from electronic medical records in a de-identified form. Our institutional electronic medical records contain the data of more than 4 million patients with more than 900 million laboratory findings and 200 million prescriptions. In addition to the institutional record, the system contains mortality data from outside the institution that is compiled using a unique personal identification number updated from the National Population Registry of the Korea National Statistical Office. Using an extracted preoperative evaluation sheet, independent investigators who were blinded to patient mortality organized the relevant preoperative variables, including demographic data, underlying disease, and laboratory testing. The SOFA score was automatically extracted from the ICU chart.

For this study, we selected patients who were transferred to the ICU and evaluated using the SOFA score immediately after surgery. Since our institutional protocol was to re-evaluate the SOFA score at midnight, we excluded patients without a second SOFA score at midnight or those who stayed in the ICU for longer than 24 h. The discharge from ICU was delayed at the discretion of the attending clinician in case of mentality change, unstable vital sign, or sign of postoperative bleeding. We initially stratified the study patients into four groups according to the quartile of the SOFA score as assessed at the time of arrival to the ICU. We divided the patients according to changes in the SOFA score. The patients were grouped into the worsened group when the follow-up SOFA score increased compared with the initial value and into the stable group when the value was unchanged or decreased.

### 2.2. Definitions and Study Endpoints

The SOFA score upon ICU admission was estimated by assessing components of the respiratory, coagulation, liver, cardiovascular, and central nervous systems and renal parameters. Assessment of the SOFA score has been well-described in a previous study [7]. According to our institutional protocol, the SOFA score was evaluated at the arrival to ICU after surgery and was followed up at midnight. We calculated the Charlson Comorbidity Index based on a preoperative diagnosis of the International Classification of Diseases-10 codes [12]. A perioperative adverse cardiac event (PACE) was defined as a composite of myocardial infarction, coronary revascularization, congestive heart failure, arrhythmic attack, acute pulmonary embolism, cardiac arrest, or stroke [13]. Postoperative pulmonary complications (PPCs) were curated according to postoperative diagnosis or imaging findings of acute respiratory distress syndrome, atelectasis, pleural effusion, pulmonary edema, pneumothorax, pneumonia, or aspiration pneumonitis [14]. Acute kidney injury (AKI) was defined according to postoperative creatinine level in the Kidney Disease: Improving Global Outcomes clinical guidelines [15]. We stratified the risk of non-cardiac surgery according to the European Society of Cardiology/European Society of Anesthesiology guidelines [16].

The primary endpoint was mortality during the three-year follow-up period. The secondary endpoints were mortalities after one year and 30 days, any PACE or PPC that occurred during the first 30 days, and postoperative AKI. Additionally, we used a machine learning technique to identify variables associated with worsening SOFA score during the first day in the ICU.

### 2.3. Statistical Analysis

The categorical variables of each group were presented as numbers and percentages and compared using a chi-square or Fisher’s exact test. The continuous variables were presented as mean ± standard deviation or median with interquartile range (IQR) as applicable, and they were compared with the t-test or the Mann–Whitney test. The mortalities were compared using a Cox regression analysis, and other outcomes were analyzed with logistic regression analysis. The results of the Cox regression analysis were reported as hazard ratio (HR) with 95% confidence interval (CI), and odds ratio (OR) was calculated for the logistic regression analysis. A multivariable adjustment was conducted by sex, age, diabetes, and peripheral arterial occlusive disease, and we also conducted a rigorous adjustment to further reduce differences in baseline characteristics with inverse probability weighting (IPW) using the propensity score for all relevant variables [17]. In this method, weights for patients with worsened SOFA scores were the inverse of the propensity score and weights for patients with stable SOFA scores. We also generated Kaplan–Meier curves for mortalities and compared then with the log-rank test. Based on the sample size, the power of our analysis was 0.81 when the HR was 1.22, and it was 0.95 when the HR was 1.30 [18]. For sensitivity analysis, we calculated the effects of unmeasured confounding factors. We evaluated the significance of the observed association between worsened SOFA score and mortality while assuming that the prevalence of unmeasured confounding factors was 40% [19]. We evaluated perioperative factors that were related to worsened SOFA using the machine learning technique with an extreme gradient boosting (XGB) algorithm, which is a decision tree-based ensemble model using the gradient boosting framework and Shapley value framework [20]. The feature importance was presented in a Shapley additive explanations (SHAP) summary plot. Analyses in this study were performed by R 4.1.0 (Vienna, Austria; http://www.R-project.org/, accessed on 24 December 2021).

## 3. Results

From a total of 203,787 patients in the SMC-NoCop registry, 66,213 (32.5%) with SOFA scores estimated immediately after surgery were selected. We excluded 48,499 patients without a second SOFA score or who stayed in the ICU for longer than 24 h; 17,714/203,787 (8.9%) patients were finally enrolled in the study. The median duration of ICU stay was 19.9 (IQR: 17.5–22.0) hours. The median values of the SOFA scores were 1 (IQR: 0–2) at ICU admission and 0 (IQR: 0–1) at the midnight follow-up. The median duration between the first and follow-up SOFA scores was 7.1 (IQR: 5.0–9.4) hours. The baseline characteristics and outcomes of the groups according to the quartile of the SOFA score upon arrival at the ICU are summarized in Table 1. The mortality during the three-year follow-up period tended to increase with a higher SOFA quartile (11.7%, 11.8%, 15.1%, and 17.8%, respectively). This tendency was consistently and clearly observed during shorter-term follow-up outcomes, such as in the mortalities at the 1-year and 30-day follow-ups, the PACE and PPC values during the 30-day follow-up, and the postoperative AKI values.

**Figure 1:**
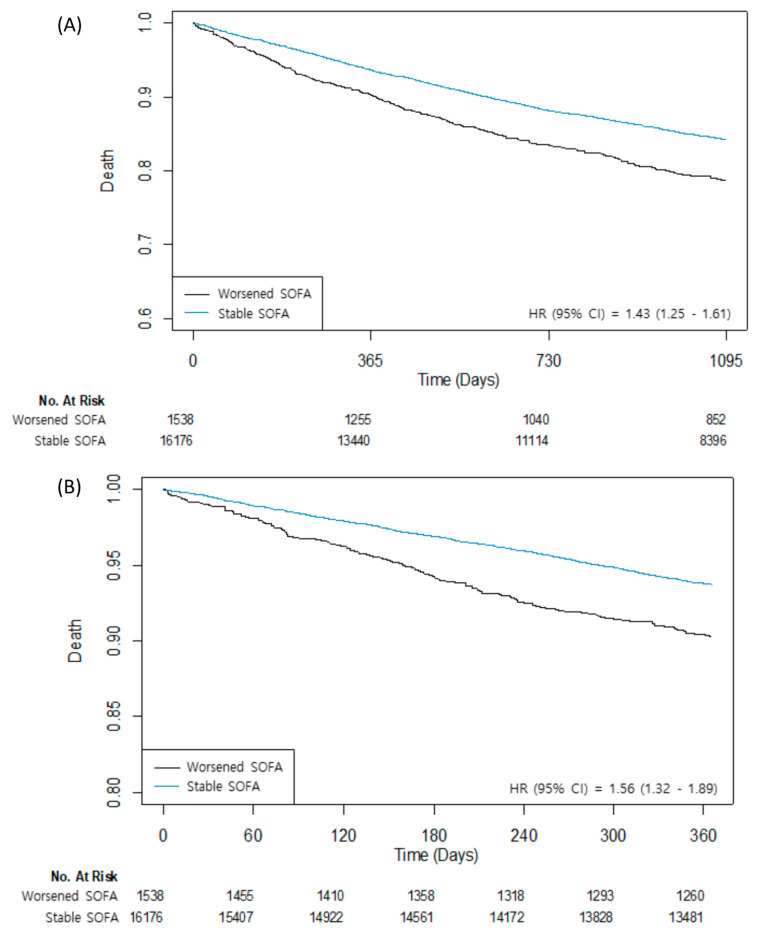
Kaplan–Meier curves for (**A**) three-year mortality and (**B**) one-year mortality.

According to the change in the follow-up SOFA score at midnight, the patients were divided into two groups: 16,176 (91.3%) in the stable group and 1538 (8.7%) in the worsened group (Table 2). The median value of the SOFA scores at ICU admission was 1 (IQR: 0–2) for both groups, but the median values of the follow-up SOFA scores were 0 (IQR: 0–1) for the stable group and 3 (IQR: 1–3) for the worsened group. Patients in the worsened group tended to be male, older, and have a higher number of comorbidities. The median duration of the follow-up period for the three-year mortality was 1095 (IQR: 573–1095) days. The mortality during the three-year follow-up was 13.7% (2421/17,714). After an IPW adjustment, the worsened group showed a significantly higher risk of three-year mortality (13.2% vs. 18.6%; HR: 1.26; 95% CI: 1.10–1.43; *p* < 0.001) (Table 3). The risks of one-year and three-year mortalities were also significantly higher for the worsened group (5.9% vs. 9.2%; HR: 1.38; 95% CI: 1.15–1.66; *p* < 0.001 and 0.4% vs. 1.0%; HR: 1.88; 95% CI: 1.05–3.37; *p* = 0.034) (Figure 1). Postoperative complications consistently showed a higher risk in the worsened group (9.5% vs. 13.6%; HR: 1.23; 95% CI: 1.04–1.45; *p* < 0.001 for PACE, 22.1% vs. 27.0%; HR: 1.26; 95% CI: 1.12–1.42; *p* < 0.001 for PPC, and 3.8% vs. 9.1%; HR: 2.13; 95% CI: 1.73–2.60; *p* < 0.001 for AKI). The observed association between a worsened SOFA score and three-year mortality was maintained as significant under any circumstance regardless of unmeasured confounding factors (Supplementary Table S1).

**Figure 2:**
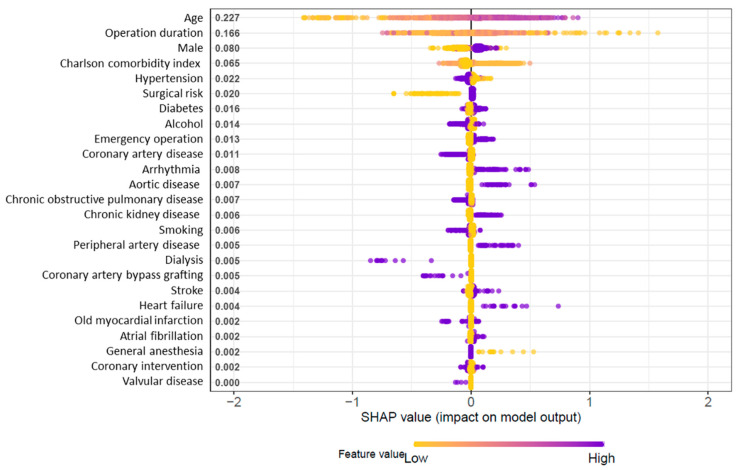
The Shapley additive explanations (SHAP) summary plot representing the results of the extreme gradient boosting (XGB) algorithm of machine learning technique.

The results of the machine learning technique with the XGB model are presented in the SHAP summary plot in which the variables are arranged in descending order by their contributions to the outcome (Figure 2). A dot marked on the variable line shows one patient and the effect of the variable on the outcome. A SHAP value greater than zero in the right section indicates an increased risk of the outcome of mortality. The variables of age, operation duration, and sex showed the largest contributions to worsened SOFA score.

## 4. Discussion

The SOFA score upon ICU admission was associated with mortality and postoperative complications in patients who were transferred to the ICU immediately after non-cardiac surgery and who stayed for less than one day. Patients with worsened SOFA scores at the midnight follow-up showed higher risk of mortality and complications compared with those with stable SOFA scores. The variables most highly associated with worsening of the SOFA score were age, operation duration, and sex.

Surgical treatment is more actively considered for an older population with various comorbidities, which contributes to an increasing number of patients who require postoperative surveillance in the ICU even for a short period [21]. Although adverse events during routine postoperative monitoring are reported to be relatively low and often resolve spontaneously, ICU admission is inevitable for patients with a comorbid status. Given that unnecessary ICU stay causes a socioeconomic burden, a reliable method to evaluate the safety of discharge from a short stay in the ICU is needed. The primary concern in these patients is the presence of organ dysfunction, which has been well-associated with increased morbidity and mortality [22,23,24].

The SOFA score was developed as an objective tool to describe and aggregate individual organ failure [5]. Numerous studies have validated its usefulness as a predictive measure with large cohorts that included both medical and surgical ICU patients [8,9,11]. Another advantage of the SOFA scoring system is that it is easy to calculate and enables fast evaluation using simple variables of major organ function derived from routine examinations. Therefore, it can be useful for a short stay in the ICU. To the best of our knowledge, this is the first study to examine the validity of the SOFA score to assess postoperative outcomes in patients admitted to the ICU for less than one day and to find that their postoperative outcomes were associated with both higher SOFA score at ICU admission and worsened SOFA score during follow-up. Clinically, our findings suggest that early identification of patients at higher risk could allow intensivists to implement a personalized treatment and establish therapeutic targets. However, the effect on mortality of patients who require a prolonged stay in the ICU remains uncertain because there are many other inter-related factors. The recommendation of specific treatment plans to improve the outcomes of these patients in the ICU is beyond the scope of our study and requires further investigation.

In addition to the one-year mortality, we also evaluated the associations for short-term mortality and complications involving different organ dysfunctions. The SOFA score showed associations with various complications, suggesting that the increased mortality may originate from the dysfunction of various organs. In fact, clinical implication of the SOFA score was initially shown for predicting the short-term mortality of sepsis or other life-threatening conditions [10,25], but has been extended to long-term outcomes of patients with trauma, acute myocardial infarction, or cardiac ICU treatment [26,27,28]. Our findings suggest that a worsened SOFA score adequately reflects organ dysfunction during the early postoperative period, which leads to increased long-term mortality. The systemic use of the SOFA score in such patients may enable early detection of acute organ dysfunction and improve long-term mortality. In addition, future studies need to evaluate the association with mortality for the simpler version of the quick SOFA score [29].

For further analysis, we used XGB algorithms for machine learning techniques to evaluate the modifiable risk factors of worsened SOFA scores. The clinical relevance should be carefully considered when interpreting the results from artificial intelligence in medicine. According to our SHAP summary plot, advanced age had a definite positive impact on increasing SOFA score, which has been suggested as a risk factor in other clinical settings as well [30,31]. The total operation time is also a well-known risk factor for postoperative outcomes but has rarely been associated with the SOFA score. Additionally, male sex showed the third highest importance, positively contributing to the prediction of worsened SOFA score. Previous studies in a sepsis cohort also reported more adverse outcomes in males [32,33], which may have been ascribed to detrimental immunological effects related to sex hormones and genetic polymorphisms [34,35]. However, the differences for males and females are inconsistent, and few studies have evaluated patients with a short stay in the ICU by sex. Therefore, further studies are needed on the associations between risk factors and SOFA score as well as on treatments that could be administered during ICU stay that could prevent or improve the SOFA score.

This study had several limitations that must be considered when interpreting our results. First, this was a retrospective study, so residual confounding factors may have affected our results despite proper statistical adjustments and a sensitivity analysis. Although all the SOFA scores were precisely extracted, the data used for this analysis were not primarily collected for this study. Therefore, assessment of the Glasgow Coma Scale might have induced some misclassification due to interobserver variability [36], but we presume that this non-differential misclassification did not bias our results. Second, the subsequent SOFA scores were routinely calculated at midnight according to the ICU protocol, regardless of the time of the first assessment. Therefore, the time interval between the initial and follow-up SOFA score was not controlled, and we could not conclude the best time interval for the second assessment of the SOFA score from this study. Third, our study included only a specific subgroup of ICU patients who stayed less than one day after non-cardiac surgery. Thus, our results cannot be generalized to other groups, such as low-risk patients, non-surgical patients or those with extended ICU stays. Lastly, we could not identify any modifiable factors that could prevent or improve the SOFA score during an ICU stay. Despite these limitations, we demonstrated an association between SOFA score and mortality in patients with a short stay in the ICU after non-cardiac surgery. Our study suggests that the SOFA score should be considered when predicting these patients’ short- and long-term prognoses and when planning effective care and therapeutic interventions.

## 5. Conclusions

The SOFA score upon ICU admission was associated with mortality and postoperative complications in patients with a stay in the ICU of less than one day after non-cardiac surgery. Patients with a worsened SOFA score at the midnight follow-up showed higher risk of mortality and complications compared with those with stable SOFA scores. The change in SOFA score may need to be considered to predict short- and long-term prognoses at discharge from the ICU.

## Figures and Tables

**Table 1:** Baseline characteristics and outcomes according to quartile values of SOFA score immediately after surgery.

	1st Quartile = 0 (N = 5163)	2nd Quartile = 1 (N = 5286)	3rd Quartile = 2 (N = 3626)	4th Quartile ≥ 3 (N = 3639)
Immediate SOFA score	0 (0–0)	1 (1–1)	2 (2–2)	4 (3–5)
Follow-up SOFA score	0 (0–0)	0 (0–0)	1 (0–1)	1 (0–2)
ICU stay, hours	19.6 (±3.0)	19.6 (±3.0)	19.5 (±3.1)	19.0 (±3.3)
Preoperative ICU	34 (0.7)	17 (0.3)	17 (0.5)	47 (1.3)
Scheduled ICU admission	3774 (73.1)	2723 (51.5)	2163 (59.7)	2984 (82.0)
Male	2345 (45.4)	2904 (54.9)	2160 (59.6)	2442 (67.1)
Age	55.1 (±14.1)	58.8 (±12.8)	60.7 (±12.0)	62.7 (±12.7)
Hypertension	1456 (28.2)	1738 (32.9)	1393 (38.4)	1597 (43.9)
Diabetes	684 (13.2)	822 (15.6)	714 (19.7)	841 (23.1)
Current alcohol	1114 (21.6)	928 (17.6)	642 (17.7)	613 (16.8)
Current smoking	493 (9.5)	336 (6.4)	277 (7.6)	272 (7.5)
Chronic kidney disease	17 (0.3)	336 (6.4)	277 (7.6)	272 (7.5)
Dialysis	0	1 (0.0)	3 (0.1)	67 (1.8)
Previous disease				
Charlson comorbidity index	0.26 (±0.66)	0.29 (±0.75)	0.35 (±0.85)	0.53 (±1.19)
Stroke	181 (3.5)	194 (3.7)	152 (4.2)	225 (6.2)
Coronary artery disease	165 (3.2)	186 (3.5)	140 (3.9)	215(5.9)
Heart failure	18 (0.3)	22 (0.4)	25 (0.7)	75 (2.1)
Arrhythmia	87 (1.7)	139 (2.6)	100 (2.8)	164 (4.5)
Peripheral artery disease	68 (1.3)	61 (1.2)	39 (1.1)	63 (1.7)
Aortic disease	39 (0.8)	44 (0.8)	50 (1.4)	119 (3.3)
Valvular heart disease	5 (0.1)	13 (0.2)	7 (0.2)	16 (0.4)
Chronic obstructive pulmonary disease	131 (2.5)	213 (4.0)	193 (5.3)	200 (5.5)
Operative variables				
Intermediate-to-high surgical risk	5019 (97.2)	5163 (97.7)	3530 (97.4)	3424 (94.1)
General anesthesia	5147 (99.7)	5274 (99.8)	3611 (99.6)	3605 (99.1)
Emergency operation	335 (6.5)	247 (4.7)	181 (5.0)	356 (9.8)
Operation duration, min	194 (±106)	190 (±100)	211 (±118)	242 (±133)
Operation type				
Neuroendocrine	31 (0.6)	23 (0.4)	16 (0.4)	24 (0.7)
Lung	1148 (22.2)	2395 (45.3)	1400 (38.6)	725 (19.9)
Head and Neck	2509 (48.6)	1429 (27.0)	782 (21.6)	608 (16.7)
Breast	11 (0.2)	10 (0.2)	5 (0.1)	1 (0.0)
Stomach	170 (3.3)	120 (2.3)	94 (2.6)	132 (3.6)
Hepatobiliary	441 (8.5)	456 (8.6)	680 (18.8)	940 (25.8)
Colorectal	242 (4.7)	189 (3.6)	148 (4.1)	257 (7.1)
Urology	46 (0.9)	60 (1.1)	58 (1.6)	112 (3.1)
Gynecology	39 (0.8)	37 (0.7)	39 (1.1)	45 (1.2)
Bone and Skin, etc.	526 (10.2)	567 (10.7)	404 (11.1)	795 (21.8)
Outcomes				
3-year mortality	603 (11.7)	622 (11.8)	548 (15.1)	648 (17.8)
1-year mortality	256 (5.0)	265 (5.0)	240 (6.6)	335 (9.2)
30-day mortality	8 (0.2)	14 (0.3)	17 (0.5)	46 (1.3)
PACE	362 (7.0)	484 (9.2)	374 (10.3)	522 (14.3)
PPC	808 (15.6)	1133 (21.4)	933 (25.7)	1120 (30.8)
Acute kidney injury, any	96 (1.5)	150 (2.8)	142 (3.9)	380 (10.4)
Stage 1	63 (1.2)	139 (2.6)	126 (3.5)	306 (8.4)
Stage 2	10 (0.2)	7 (0.1)	14 (0.4)	44 (1.2)
Stage 3	3 (0.1)	4 (0.1)	2 (0.1)	30 (0.8)

Data are presented as *n* (%), mean (±standard deviation), or median (interquartile). Surgical risk was stratified according to 2014 European Society of Cardiology/European Society of Anaesthesiology guidelines. SOFA, sequential organ failure assessment; ICU, intensive care unit; PACE, perioperative adverse cardiac events; PPC, postoperative pulmonary complication.

**Table 2:** Baseline characteristics according to SOFA score change during short ICU stay.

	Stable SOFA (N = 16,176)	Worsened SOFA (N = 1538)	*p*-Value	ASD before IPW	ASD after IPW
Immediate SOFA score	1 (0-2)	1 (0-2)	<0.001	35.5	
Follow-up SOFA score	0 (0-1)	3 (1-3)	<0.001	>99	
ICU stay, hours	19.5 (±3.1)	19.5 (±3.3)	0.56	1.5	1.6
Preoperative ICU	17 (1.1)	98 (0.6)	0.03	5.4	2.3
Scheduled ICU admission	928 (60.3)	10716 (66.2)	<0.001	12.3	0.9
Male	8886 (54.9)	965 (62.7)	<0.001	15.9	0.7
Age	58.6 (±13.4)	62.0 (±12.7)	<0.001	26.7	0.6
Hypertension	5599 (34.6)	585 (38.0)	0.01	7.1	0.2
Diabetes	2739 (16.9)	322 (20.9)	<0.001	10.2	<0.01
Current alcohol	3034 (18.8)	263 (17.1)	0.12	4.3	1.4
Current smoking	1248 (7.7)	130 (8.5)	0.33	2.7	0.8
Chronic kidney disease	310 (1.9)	46 (3.0)	<0.001	6.9	0.4
Dialysis	67 (0.4)	4 (0.3)	0.48	2.7	3.2
Previous disease					
Charlson comorbidity index	0.34 (±0.85)	0.42 (±1.00)	<0.001	9.5	5.4
Stroke	669 (4.1)	83 (5.4)	0.02	5.9	0.7
Coronary artery disease	642 (4.0)	64 (4.2)	0.76	1	0.3
Heart failure	124 (0.8)	16 (1.0)	0.31	2.9	1.9
Arrhythmia	425 (2.6)	65 (4.2)	<0.001	8.8	0.2
Peripheral artery disease	192 (1.2)	39 (2.5)	<0.001	10	0.1
Aortic disease	211 (1.3)	41 (2.7)	<0.001	9.8	0.9
Valvular heart disease	39 (0.2)	2 (0.1)	0.56	2.6	0.7
Chronic obstructive pulmonary disease	666 (4.1)	71 (4.6)	0.38	2.4	0.5
Operative variables					
Intermediate-to-high surgical risk	15,632 (96.6)	1504 (97.8)	0.02	7	0.9
General anesthesia	16,110 (99.6)	1527 (99.3)	0.12	4.1	1.4
Emergency operation	1005 (6.2)	114 (7.4)	0.07	4.8	0.3
Operation duration, min	206 (±115)	209 (±110)	0.36	2.5	1.1
Operation type			<0.001	34.3	24.4
Neuroendocrine	86 (0.5)	8 (0.5)			
Lung	5116 (31.6)	552 (35.9)			
Head and Neck	5055 (31.2)	273 (17.8)			
Breast	26 (0.2)	1 (0.1)			
Stomach	481 (3.0)	35 (2.3)			
Hepatobiliary	2213 (13.7)	304 (19.8)			
Colorectal	761 (4.7)	75 (4.9)			
Urology	252 (1.6)	24 (1.6)			
Gynecology	144 (0.9)	16 (1.0)			
Bone and Skin, etc.	2042 (12.6)	250 (16.3)			

Data are presented as *n* (%), mean (±standard deviation), or median (interquartile). Surgical risk was stratified according to 2014 European Society of Cardiology/European Society of Anaesthesiology guidelines. SOFA, sequential organ failure assessment; ICU, intensive care unit; ASD, absolute standardized mean difference; IPW, inverse probability weighting.

**Table 3:** Mortalities according to perioperative adverse cardiac event (PACE).

	Stable SOFA (N = 16,176)	Worsened SOFA (N = 1538)	Unadjusted HR or OR (95% CI)	*p*-Value	Adjusted HR or OR (95% CI)	*p*-Value	IPW Adjusted HR or OR (95% CI)	*p*-Value
3-year mortality	2135 (13.2)	286 (18.6)	1.42 (1.26–1.61)	<0.001	1.32 (1.16–1.49)	<0.001	1.26 (1.10–1.43)	<0.001
1-year mortality	955 (5.9)	141 (9.2)	1.57 (1.32–1.88)	<0.001	1.45 (1.22–1.73)	<0.001	1.38 (1.15–1.66)	<0.001
30-day mortality	70 (0.4)	15 (1.0)	2.26 (1.29–3.95)	0.004	2.11 (1.20–3.69)	0.01	1.88 (1.05–3.37)	0.034
PACE *	1533 (9.5)	209 (13.6)	1.50 (1.28–1.75)	<0.001	1.28 (1.09–1.50)	<0.001	1.23 (1.04–1.45)	<0.001
PPC *	3578 (22.1)	416 (27.0)	1.31 (1.59–1.47)	<0.001	1.22 (1.08–1.38)	<0.001	1.26 (1.12–1.42)	<0.001
Acute kidney injury, any *	608 (3.8)	140 (9.1)	2.56 (2.11–3.10)	<0.001	2.25 (1.84–2.72)	<0.001	2.13 (1.73–2.60)	<0.001
Stage 1	517 (3.2)	117 (7.6)						
Stage 2	62 (0.4)	13 (0.8)						
Stage 3	29 (0.2)	10 (0.7)						

Data are presented as *n* (%). The multivariable analysis retained male, age, diabetes, and peripheral arterial occlusive disease. IPW adjustment retained all relevant variables. Risk of mortality was presented as HR and the other outcomes * are presented with OR. HR, hazard ratio; OR, odds ratio; CI, confidence interval; IPW, inverse probability weighting; PACE, perioperative adverse cardiac events; PPC, postoperative pulmonary complication.

## Data Availability

The data presented in this study are available on request from the corresponding author. The data are not publicly available due to institutional restriction.

## Supplementary Materials

The following supporting information can be downloaded at: https://www.mdpi.com/article/10.3390/jcm11195865/s1. Table S1: Effect of an unmeasured confounder on hazard ratio of worsened SOFA score for three-year mortality after IPW adjustment.
