# Supplementary material for: Association between Mortality and Sequential Organ Failure Assessment Score during a Short Stay in the Intensive Care Unit after Non-Cardiac Surgery
Source: J Clin Med. 2022 Oct 4;11(19):5865. doi: 10.3390/jcm11195865 (PMC9573186; doi:10.3390/jcm11195865)
Supplement: Supplementary file 1 [file jcm-11-05865-s001.zip › jcm-1913216-supplementary.pdf]

Table S1. Effect of an unmeasured confounder on hazard ratio of worsened SOFA score for three-year mortality after IPW adjustment.

		$OR_{ZY\|X}$					
		1.5	2	2.5	3	3.5	4
	0.3	1.59 (1.40–1.80)	1.71 (1.51–1.94)	1.83 (1.61–2.07)	1.91 (1.69–2.17)	2.06 (1.81–2.33)	2.09 (1.84–2.37)
	0.4	1.55 (1.37–1.76)	1.64 (1.44–1.85)	1.73 (1.53–1.96)	1.81 (1.59–2.05)	1.88 (1.66–2.13)	1.93 (1.71–2.19)
OR_{zx}	0.5	1.51 (1.34–1.71)	1.59 (1.40–1.80)	1.66 (1.46–1.88)	1.71 (1.51–1.94)	1.78 (1.58–2.02)	1.80 (1.59–2.04)
	0.6	1.49 (1.32–1.69)	1.54 (1.36–1.75)	1.59 (1.41–1.80)	1.63 (1.44–1.84)	1.67 (1.418–1.89)	1.70 (1.50–1.92)
	0.7	1.47 (1.30–1.67)	1.51 (1.33–1.71)	1.53 (1.35–1.73)	1.56 (1.38–1.76)	1.59 (1.41–1.80)	1.62 (1.43–1.84)

Prevalence of unmeasured confounder = 40%. Numbers represent HRs (including 95% CIs). HR, hazard ratio; X: dichotomous exposure measure, y dichotomous outcome measure, z : potential dichotomous confounder. ORZX indicates the association (OR) between the unmeasured confounder and worsened SOFA score. ORZY|X indicates the association (OR) between the unmeasured confounder and three-year mortality.
